# Aerosol Transmission of the Pandemic SARS-CoV-2 and Influenza A Virus Was Blocked by Negative Ions

**DOI:** 10.3389/fcimb.2022.897416

**Published:** 2022-04-29

**Authors:** Cheng Zhang, Huan Cui, Chunmao Zhang, Zhaoliang Chen, Xinyun Jiang, Jun Liu, Zhonghai Wan, Jiping Li, Juxiang Liu, Yuwei Gao, Ningyi Jin, Zhendong Guo

**Affiliations:** ^1^ Changchun Veterinary Research Institute, Chinese Academy of Agricultural Sciences, Changchun, China; ^2^ College of Veterinary Medicine, Hebei Agricultural University, Baoding, China; ^3^ College of Veterinary Medicine, Jilin University, Changchun, China

**Keywords:** SARS-CoV-2, influenza A viruses, disinfection, aerosol transmission, negative ions

## Abstract

The pandemic of respiratory diseases, such as coronavirus disease 2019 (COVID-19) and influenza, has imposed significant public health and economic burdens on the world. Wearing masks is an effective way to cut off the spread of the respiratory virus. However, due to cultural differences and uncomfortable wearing experiences, not everyone is willing to wear masks; there is an urgent need to find alternatives to masks. In this study, we tested the disinfection effect of a portable ionizer on pandemic severe acute respiratory syndrome coronavirus 2 (SARS-CoV-2) (strain V34) and influenza A virus (strain CA04). Negative ions significantly reduced the concentration of particulate matter in the air above and effectively disinfected viruses stuck to the solid plate at the level of both nucleic acid and virus titer. The disinfection efficiency was >99.8% after 1-h exposure. Moreover, negative ions effectively disinfected aerosolized viruses; the disinfection efficiency was more than 87.77% after purification for 10 min. Furthermore, negative ions had a significant protective effect on susceptible animals exposed to viral aerosols. When the negative ionizer was switched from off to on, the inhalation 50% infective dose (ID_50_) for golden hamsters challenged with SARS-CoV-2 rose from 9.878 median tissue culture infective dose (TCID_50_) [95% confidence interval (CI), 6.727–14.013 TCID_50_] to 43.891 TCID_50_ (95% CI, 29.31–76.983 TCID_50_), and the inhalation ID_50_ for guinea pigs challenged with influenza A virus rose from 6.696 TCID_50_ (95% CI, 3.251–9.601 TCID_50_) to 28.284 TCID_50_ (95% CI, 19.705–40.599 TCID_50_). In the experiment of transmission between susceptible animals, negative ions 100% inhibited the aerosol transmission of SARS-CoV-2 and influenza A virus. Finally, we tested the safety of negative ion exposure. Balb/c mice exposed to negative ions for 4 weeks showed no abnormalities in body weight, blood routine analysis, and lung pathology. Our study demonstrates that air ions can be used as a safe and effective means of blocking respiratory virus transmission and contribute to pandemic prevention and control.

## Introduction

Respiratory viruses continue to pose heavy burdens on global health and economics. Since its first found in December 2019, severe acute respiratory syndrome coronavirus 2 (SARS-CoV-2) has spread globally for more than 2 years, causing 452,201,564 confirmed cases of coronavirus disease 2019 (COVID-19) and 6,029,852 deaths (World Health Organization). Influenza caused annual seasonal epidemics, sporadic and unpredictable global pandemic outbreaks. The WHO estimates that annual epidemics of influenza result in ~1 billion infections, 3–5 million cases of severe illness, and 300,000–500,000 deaths (Krammer et al., 2018).

Airborne transmission is one of the important transmission routes of respiratory viruses (Zhou et al., 2018; Ram et al., 2021). Infected patients could produce droplets or aerosols when breathing, coughing, sneezing, vomiting, etc. (Stilianakis and Drossinos, 2010; Comber et al., 2021). Ma et al. (2021) found that COVID-19 patients exhaled millions of SARS-CoV-2 RNA copies per hour, and the air of patient wards had been proven to contain virus-laden aerosols (Guo et al., 2020; Liu et al., 2020). Yan et al. (2018) recovered infectious virus from 39% of the fine aerosols (≤5 μm) sampled from volunteers with confirmed influenza infection. Major known methodologies in place to prevent respiratory viral disease include wearing masks (Brienen et al., 2010) and vaccination (Zhu et al., 2020). However, due to cultural differences and uncomfortable wearing experiences, it is difficult to wear masks all the time. In addition, because the virus mutates quickly, the vaccine needs to be frequently updated (Kim et al., 2018), and the presence of antibodies in the body also cannot guarantee not being infected (Zhang et al., 2021). Hence, attention must be given to explore new alternative solutions such as air disinfection by negative ions to reinforce efforts in preventing the spread of respiratory viral disease.

On the one hand, negative ions can neutralize and capture small particles and dust, so that they condense and precipitate, effectively removing PM_2.5_ particulate pollutants in the air (Grinshpun et al., 2005). On the other hand, negative ions can directly disinfect pathogenic microorganisms. Negative ions gain hydrogen from surface molecules of pathogens due to electrostatic force. The elimination of hydrogen from or the disruption of the surface molecules of pathogens results in inactivation (Nunayon et al., 2022).

Studies have shown that negative ions could disinfect a wide variety of aerosolized microorganisms, including *Salmonella typhimurium* (Nunayon et al., 2022), *Staphylococcus epidermidis* (Kim et al., 2011; Lee et al., 2014; Zhou et al., 2018; Nunayon et al., 2022), *Serratia marcescens* (Zhou et al., 2018), *Escherichia coli* (Huang et al., 2008; Park et al., 2009; Kim et al., 2011), *Bacillus subtilis* (Huang et al., 2008), *Aspergillus niger*, and *Aspergillus versicolor* (Huang et al., 2008). Escombe et al. (2009) found that negative air ionization prevented most airborne *Mycobacterium tuberculosis* transmission detectable by guinea pig air sampling. Gast et al. (1999) found that negative ions reduced the airborne transmission of *Salmonella enteritidis* to chicks. However, previous studies on aerosolized microorganism disinfection by negative ions mainly focused on bacteria; very few studies determined the disinfection performance of negative ions against aerosolized viruses. Hyun et al. (2017) found that bipolar ions showed a disinfection effect on the filtration of aerosolized bacteriophage MS2, and the antiviral efficiency of bipolar ions was higher than that of positive air ions. Hagbom et al. (2015) found that negative ions could inactivate calicivirus, rotavirus, and influenza virus and 100% (4/4) prevent airborne transmission of influenza A virus between guinea pigs. The reduction of transmission of Newcastle disease virus by negative ions was also reported previously (Estola et al., 1979; Mitchell and King, 1994). However, none of them evaluated the effects of negative ions on blocking coronavirus infection and transmission in animal experimental setups.

In this study, the disinfection effects of negative ions on two pandemic respiratory viruses (SARS-CoV-2 and influenza A virus) were experimentally investigated. The disinfection efficiency at two heights (30 and 50 cm) was studied. Furthermore, we also tested the performance of negative ions in preventing infection by viral aerosol exposure and blocking natural aerosol transmission. Finally, the safety of negative ions was evaluated by mouse exposure experiment, and concern on ozone emission is also discussed.

## Materials and Methods

### Ethics Statement

All experiments with animals were performed in strict accordance with the guidelines on animal welfare of the World Organization for Animal Health. Experimental protocols involving animals were approved by the Animal Care and Use Committee of the Changchun Veterinary Institute (approval number: SMKX-20200915–11). All experiments with SARS-CoV-2 and influenza A virus were performed in a Biosafety Level 3 laboratory.

### Viruses

Two pandemic viruses, SARS-CoV-2 and influenza A virus, were selected as test pathogens. The human SARS-CoV-2 isolate was BetaCoV/Beijing/IME-BJ05-2020 (abbreviated as V34), and the human influenza A virus isolate was the 2009 pandemic H1N1 A/California/04/2009 (abbreviated as CA04). SARS-CoV-2 and influenza A virus were grown in VeroE6 cells and Madin-Darby Canine Kidney (MDCK) cells [purchased from American Type Culture Collection (ATCC)], respectively. SARS-CoV-2 and influenza A virus were titrated in the VeroE6 cells and MDCK cells, respectively, to determine the median tissue culture infective dose (TCID_50_) using the Reed–Muench method. They were all stored at -80°C before use.

### Negative Ionizers

The negative ionizers were portable and wearable products made of brush-type ion-generating emission tips with power packs in a custom-made plastic casing (Model EKT-001; AIRCLEAN Electronic and Technology Co. Ltd., Beijing, China). When worn around the neck, the ion-generating emission tips was usually 30–50 cm below the head (**Figure S1**). Therefore, we chose 30 and 50 cm as test heights in the experiment. The concentration of negative ions was determined by an air counter (Model LD-FY1; Lanende Inc., Shandong, China) of which the ion count capacity can reach up to 50 million ions/cm^3^.

### Particulate Matter Purification

Two laser particle counters (Model 9306, TSI Inc., MN, USA) were used to monitor the concentration of particulate matter (PM) in the air. They were placed 30 and 50 cm above the ionizer, respectively. Data were recorded every 10 s. The sampling rate of the laser particle counter was 2.83 L/min, and the air sampling time was 6 s. We first switch on the laser particle counters. After collecting approximately 50 background data, then the negative ionizer was switched on.

### Disinfection of Fixed Virus

A piece of gelatin filter (diameter 25 mm; SKC, Inc.) was placed in the middle of a sterile cell culture plate, and then 100 μl of virus suspension was dropped on the filter. After the virus suspension was absorbed and the filter adhered to the plate, the plate was placed upside down 30 or 50 cm above the ionizer. The disinfection time was set to 5, 10, 20, 40, and 60 min. After disinfection, the gelatin filter was collected, melted at 37°C, and subjected to virus titration, RNA extraction, and real-time RT-PCR.

### Disinfection of Aerosolized Virus

The disinfection effect of negative ions on aerosolized virus was evaluated in a Biosafety Level 3 glove box. A mannequin of the upper body wearing a mask is placed on one side of the glove box, and a button sampler (SKC, Inc.) is placed next to the head at the height of the mouth. The negative ionizer was 30 or 50 cm below the mouth of the mannequin. A 24-jet Collison-type nebulizer (BGI, Inc.) was used to generate viral aerosols, and it was placed at the same height as the mouth and at a horizontal distance of 50 cm. The generator outlet is facing the mouth and nose position of the mannequin.

Viruses were diluted to 500 TCID_50_/ml and subsequently transferred into the 24-jet Collison-type nebulizer and aerosolized. Compressed air was delivered to the nebulizer through a dry-cleaned air supply system consisting of a dryer and filter. The pressure of the compressor was maintained at 103.42 kPa. Air sampling was conducted using the button sampler loaded with a 25-mm-diameter gelatin filter. The sampling flow rate was 4.0 L/min. Air sampling was initiated at the same time as aerosol generation. After 10 min, aerosol generation and air sampling were both switched off. The gelatin filter was dissolved in 2 ml TRIzol (Thermo Fisher Scientific Inc.) and subsequently used to extract RNA for viral nucleic acid testing. The mask was cut into 1 × 1-cm pieces, and 6 of them were vortex oscillated in 1 ml TRIzol for 5 min, and then the supernatant was used to extract RNA for viral nucleic acid testing. Between each air sampling time, laboratory wipes with 75% ethanol were used for sterilizing the sampler and the mannequin.

### Viral Aerosol Exposure

To better simulate the protective effect of negative ions on virus exposure, we conducted experiments on animals exposed to viral aerosol in an animal exposure cabin. The air ionizer is mounted on the inner wall of the cabin. Five-week-old male golden hamsters and Hartley strain female guinea pigs weighing 300–350 g (Beijing Vital River Laboratory Animal Technology Co. Ltd.) were used as susceptible experimental animals for SARS-CoV-2 and influenza A virus, respectively.

Viruses were diluted to 62.5, 125, 250, 500, 10^3^, 2 × 10^3^ TCID_50_/ml, respectively, and subsequently transferred separately into the 24-jet Collison-type nebulizer and aerosolized. Two fans are installed in the exposed chamber for mixing the internal aerosols. All groups of animals (n = 10) were challenged with viral aerosol for 10 min. After exposure, the animals were placed in individually ventilated cages. Nasal washes of these animals were collected and titrated at 3 days post exposure to determine being infected or not. When carrying out the experiment with the highest concentration of virus suspension, we collected internal aerosol samples using button sampler to calculate the inhalation dose (IND) of animals. IND was calculated as: *IND* = (S_v_ * V_a_)/V_s_, where *S_v_* is the virus titer collected by button sampler, *V_a_* is the respiratory dose per minute of an animal, and *V_s_* is the sampling rate of the button sampler. The inhalation dose of other groups was calculated in proportion to the concentration of the pre-spray virus suspension concentrations.

### Transmission Evaluation

Five-week-old male golden hamsters and Hartley strain female guinea pigs weighing 300–350 g were used as susceptible experimental animals for SARS-CoV-2 and influenza A virus, respectively. Six donor animals per group were intranasally inoculated with 10^5^ TCID_50_ of the corresponding virus. At 24 h post inoculation, 3 donors per group were transferred to a new cage and cohoused with three naive animals for the direct contact transmission studies, and another 3 donors per group were transferred to a wire-frame cage adjacent to another 3 naive animals for the aerosol transmission studies. The distance between the donor animals and the aerosol-contact animals was 5.0 cm. In the direct contact transmission experiment, the air ionizer is mounted on the inner wall of the cage. In the aerosol transmission experiment, the air ionizer is placed between the donor and the naive animals. Nasal washes of these animals were collected and titrated at 1, 3, 5, and 7 days post inoculation or exposure.

### Viral Nucleic Acid Testing

RNA was extracted using the QIAamp Viral RNA Mini kit (Qiagen, Germantown, MD, USA) and detected using the One Step PrimeScript™ RT-PCR Kit (TaKaRa, Japan) according to the manufacturers’ protocol. Quantitative real-time PCR (Q-RT-PCR) assays were performed by using a set of primers and probes (For V34, Forward: 5′-GGGGAACTTCTCCTGCTAGAAT-3′; Reverse: 5′-CAGACATTTTGCTCTCAAGCTG-3′; Probe: FAM-5′-TTGCTGCTGCTTGACAGATT-3′-TAMRA; Targeting regions: *N gene*. For CA04, Forward: 5′-CATTGAAGGGGGGTGGACAG-3′; Reverse: 5′-GGTGGTTGAACTCTTTACCTACTGC-3′; Probe: FAM-5′-ACCATCAAAATGAGCAGGGGTCAGG-3′-TAMRA; Targeting regions: *HA gene*], and the PCRs were run on the ABI 7500 System (Thermo Fisher Scientific, Waltham, MA, USA). Standard curves were calibrated for virus copy number using plasmids containing a cDNA copy of the Q-RT-PCR target amplicon.

### Safety Evaluation

To verify the safety of exposure to negative ions, 20 female Balb/c mice (6-week-old, weighing 18–20 g) were purchased from Beijing Vital River Laboratory Animal Technology Co., Ltd. These mice were fed in the IVC system and given normal water and feed during the experiment. These Balb/c mice were separated into two groups, each with 10 mice. One group served as the control, while the other served as the negative ion exposure group. The air ionizer is mounted on the inner wall of the cage. The body weight of mice was monitored daily for 30 days. On day 30, blood samples of these mice were collected for blood routine analysis according to the manual of automatic blood cell analysis instrument (BT-3200, Better, China), and the lungs of mice were fixed with formalin, embedded in paraffin, and stained with hematoxylin and eosin (HE).

### Statistical Analysis

Statistically significant differences were determined using one-way analysis of variance (ANOVA) with GraphPad Prism software (San Diego, CA, USA). All of the assays were run in triplicate and are representative of at least 3 separate experiments. The error bars represent the standard deviation. *P* values <0.05 indicated significant differences. The 50% infective dose (ID_50_) was evaluated by probit analysis. The independent variable for the probit analysis was dose, and the dependent variable was infection rate.

## Results

### Particulate Matter Purification by Negative Ions

**Figure 1:**
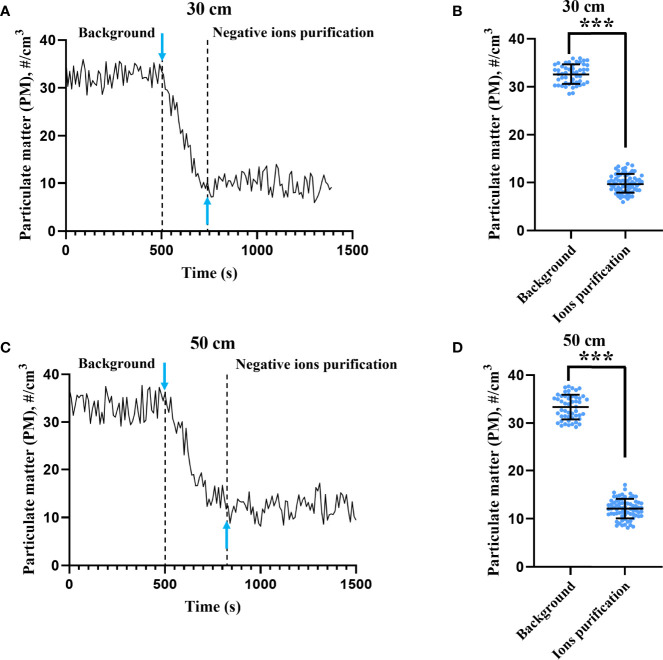
Particulate matter purification effect by negative ions. The concentrations of particulate matter (PM) were monitored by two laser particle counters placed 30 **(A)** and 50 cm **(C)** above the ionizer. Downward arrow means that the ionizer was switched on at that moment. Upward arrow indicates the moment when the concentration of PM first dropped to the mean value after negative ion purification. The PM concentrations before and after negative ion purification at 30 **(B)** and 50 cm **(D)** height were also compared. The bar means: Mean ± SD. *** means *P* value <0.001.

The ion concentrations at 30 and 50 cm above the negative ionizer were 1.06 ± 0.08 × 10^5^ ions/cm^3^ and 5.61 ± 0.64 × 10^4^ ions/cm^3^, respectively. To test the efficiency of PM purification, laser particle counters were used to monitor the PM concentration before and after switching on the negative ionizer. As shown in **Figure 1**, negative ions exhibited significant PM purification effect at both 30- and 50-cm height. At 30-cm height (**Figures 1A, B** **)**, the PM concentrations before and after negative ion purification were 32.65 ± 2.01 particles/cm^3^ and 9.87 ± 1.93 particles/cm^3^, respectively. The purification efficiency was 69.77%. The response time to reach the average PM concentration after purification was 220 s. At 50-cm height (**Figures 1C, D** **)**, the PM concentrations before and after negative ion purification were 33.36 ± 2.56 particles/cm^3^ and 12.18 ± 2.02 particles/cm^3^, respectively. The purification efficiency was 63.49%. The response time to reach the average PM concentration after purification was 300 s.

### Fixed Virus Disinfection Efficiency of Negative Ions

**Figure 2:**
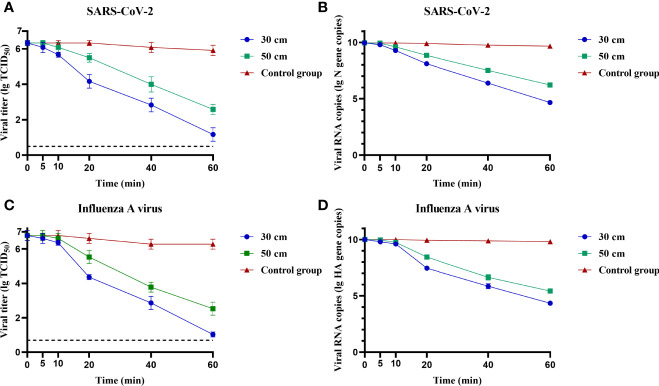
Fixed virus disinfection effect by negative ions. The disinfection effect of negative ions on fixed severe acute respiratory syndrome coronavirus 2 (SARS-CoV-2) **(A, B)** and influenza A virus **(C, D)** was analyzed at different time points and different heights. Both viral titer and RNA copies were determined. Dashed lines indicate the lower limit of virus detection. The bar means: Mean ± SD.

To determine the disinfection efficiency of negative ions on SARS-CoV-2 and influenza A virus, the virus was adhered on a cell culture plate and placed upside down 30 or 50 cm above the negative ionizer. As shown in **Figure 2**, both SARS-CoV-2 and influenza A virus were highly susceptible to negative ions. Both viral titer and RNA copies were decreased with the extension of disinfection time. The disinfection efficiencies at different time points and different heights were listed in **Table S1**. After disinfection for 1 h, the disinfection efficiencies of negative ions were more than 99.98% for SARS-CoV-2 and influenza A virus at both viral titer and RNA copy levels.

### Aerosolized Virus Disinfection Efficiency of Negative Ions

**Figure 3:**
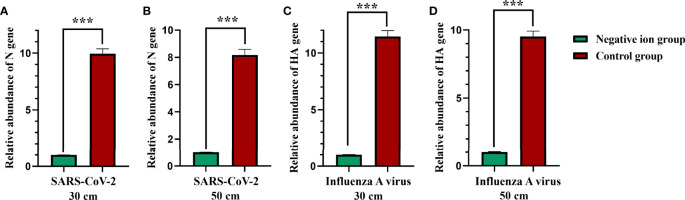
Aerosolized virus disinfection effect by negative ions. A 24-jet Collison-type nebulizer was used to aerosolize the virus suspension. A button sampler was placed at the same height, 50 cm horizontally, and used to sample the air with a flow rate of 4 L/min. The air ionizer was placed 30 **(A, C)** or 50 cm **(B, D)** below the button sampler. When the 10-min sampling is over, the relative abundance of viral RNA in the air samples was compared when the ionizer was active or inactive. The bar means: Mean ± SD. *** means P value <0.001.

We further determined the disinfection performance of negative ions against aerosolized virus. A 24-jet Collison-type nebulizer was used to aerosolize the virus suspension. A button sampler was placed at the same height, 50 cm horizontally, and used to sample the air with a flow rate of 4 L/min. The air ionizer was placed 30 or 50 cm below the button sampler. When the 10-min sampling is over, the relative abundance of viral RNA in the air samples was compared when the ionizer was active or inactive. At the height of 30 cm, the disinfection efficiencies were 89.96% and 91.27% for SARS-CoV-2 and influenza A virus, respectively (**Figures 3A, C** **)**. While at the height of 50 cm, the disinfection efficiencies were 87.77% and 89.50% for SARS-CoV-2 and influenza A virus, respectively (**Figures 3B, D** **)**. These results indicate that airborne virus can be largely inactivated by negative ions.

**Figure 4:**
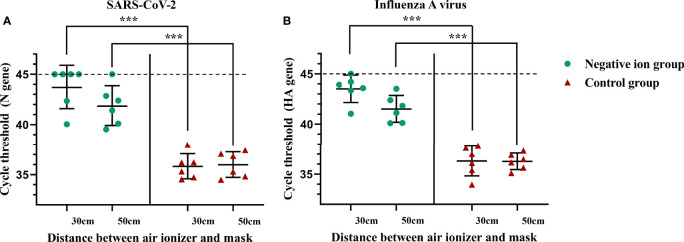
Negative ions reduce the amount of viral nucleic acid that stuck on masks. Syndrome coronavirus 2 (SARS-CoV-2) **(A)** and influenza A virus **(B)**. A mannequin of the upper body wearing a mask was facing the outlet of the nebulizer with a horizontal distance of 50 cm. The negative ionizer was 30 or 50 cm below the mouth of the mannequin. After 10 min of aerosol exposure, the mask was cut into 1 × 1-cm pieces, and six of them were vortex oscillated in 1 ml TRIzol for 5 min, and then the supernatant was used to extract RNA for viral nucleic acid testing. Dashed lines indicate no fluorescence signal to the maximum cycle (45). The bar means: Mean ± SD. *** means P value <0.001.

Furthermore, we investigated whether negative ions could reduce the amount of virus that stick on masks during the aerosolized virus exposure. A mannequin of the upper body wearing a mask is placed next to the button sampler. After 10 min of aerosol exposure, the mask was cut into 1 × 1-cm pieces and 6 of them were vortex oscillated in 1 ml TRIzol for 5 min, and then the supernatant was used to extract RNA for viral nucleic acid testing. As shown in **Figure 4**, negative ions significantly reduced the amount of viral nucleic acid stuck on masks. The disinfection efficiencies were 99.58% (7.90 log_2_ reduction) and 98.31% (5.88 log_2_ reduction) for SARS-CoV-2 at the height of 30 and 50 cm, respectively (**Figure 4A**). The disinfection efficiencies were 99.32% (7.19 log_2_ reduction) and 97.35% (5.24 log_2_ reduction) for influenza A virus at the height of 30 and 50 cm, respectively (**Figure 4B**).

### Protection Against Viral Aerosol Exposure by Negative Ions

**Figure 5:**
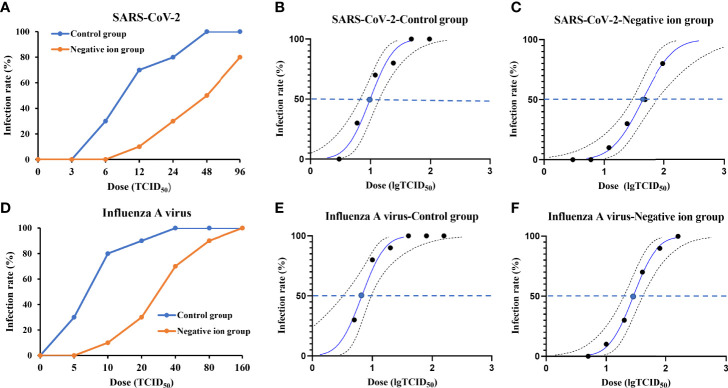
Infection rate and 50% infective dose (ID_50_) plots for animals exposed to aerosolized virus. The infection rate of animals exposed to aerosolized severe acute respiratory syndrome coronavirus 2 (SARS-CoV-2) **(A)** or influenza A virus **(D)** when the ionizer was active or inactive was determined. Panels **(B, C, E, F)** show the probit analysis to determine ID_50_ for aerosolized SARS-CoV-2 or influenza A virus exposure when the ionizer was active or inactive. Dashed lines show 95% confidence interval for the analysis.

Next, we investigated whether negative ions could protect the susceptible animals from being infected by viral aerosol exposure. Groups of animals (n = 10) were challenged with viral aerosol in an animal exposure cabin when the ionizer was active or inactive. The infection rate plots were shown in **Figures 5A, C**. Negative ions, to some extent, protected animals from aerosol infection. For SARS-CoV-2, when the ionizer was inactive, the inhalation ID_50_ for hamsters challenged with viral aerosols was estimated to be 9.878 TCID_50_, with 95% confidence interval (CI) of 6.727–14.013 TCID_50_ (**Figure 5B**). In contrast, when the ionizer was active, the ID_50_ rose to 43.891 TCID_50_, with 95% CI of 29.31–76.983 TCID_50_ (**Figure 5C**).

For influenza A virus, when the ionizer was inactive, the inhalation ID_50_ for guinea pigs challenged with viral aerosols was estimated to be 6.696 TCID_50_, with 95% CI of 3.251–9.601 TCID_50_ (**Figure 5E**). In contrast, when the ionizer was active, the ID_50_ rose to 28.284 TCID_50_, with 95% CI of 19.705–40.599 TCID_50_ (**Figure 5F**).

### Prevention of Airborne-Transmitted SARS-CoV-2 and Influenza A Virus Infection Between Animals by Negative Ions

**Figure 6:**
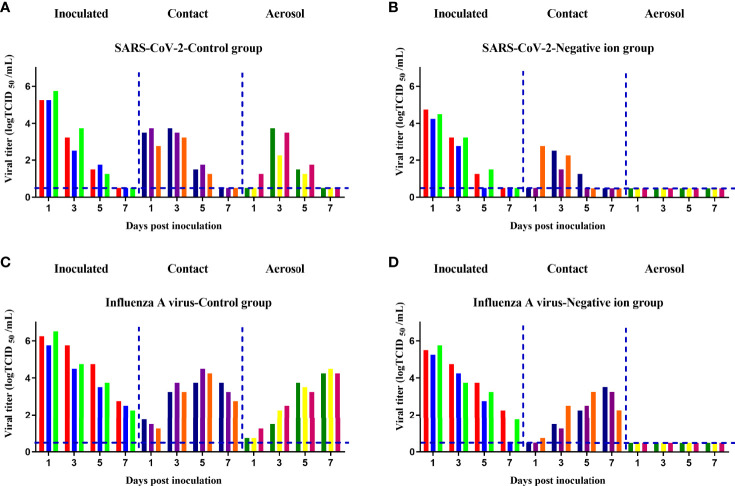
Aerosol transmission of the pandemic severe acute respiratory syndrome coronavirus 2 (SARS-CoV-2) **(A, B)** and influenza A virus **(C, D)** was blocked by negative ions. Six donor animals per group were intranasally inoculated with 105 median tissue culture infective dose (TCID50) of the corresponding virus. At 24 h post inoculation, 3 donors per group were transferred to a new cage and cohoused with three naive animals for the direct contact transmission studies, and another 3 donors per group were transferred to a wire-frame cage adjacent to another 3 naive animals for the aerosol transmission studies. Nasal washes of these animals were collected and titrated at 1, 3, 5, and 7 days post inoculation or exposure. Each color bar represents the virus titer in an individual animal. Dashed lines indicate the lower limit of virus detection.

Finally, we determined if negative ions could prevent direct contact and aerosol transmission of SARS-CoV-2 and influenza A virus between animals. In the direct contact transmission experiment, the air ionizer is mounted on the inner wall of the cage. In the aerosol transmission experiment, the air ionizer is placed between the donor and the naive animals. As shown in **Figure 6**, when the negative ionizer was inactive, 100% (3/3) of the animals in contact and aerosol transmission groups were infected (**Figures 6A, C** **)**. In contrast, when the negative ionizer was active, the ionizer protected 100% (3/3) of hamsters from SARS-CoV-2 aerosol transmission (**Figure 6B**) and 100% (3/3) of guinea pigs from influenza aerosol transmission (**Figure 6D**). Although negative ions did not block direct contact transmission, viral loads in nasal washes were reduced in both infected and transmitted animals compared to those of the control group.

### Safety Evaluation

**Figure 7:**
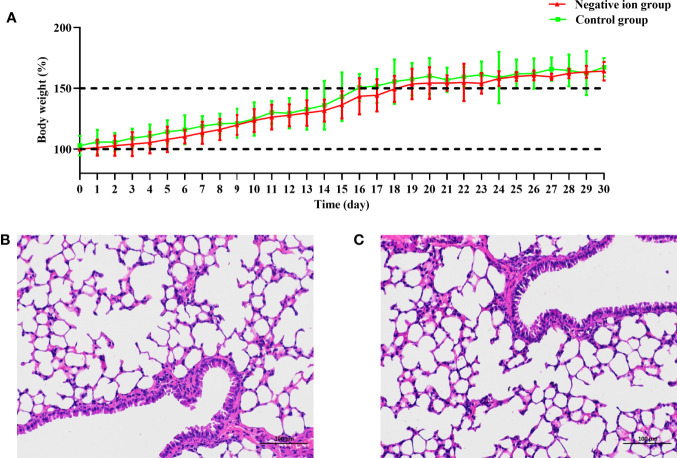
Body weight change and lung pathological observation of mice exposed to negative ions for 30 days. Ten mice were exposed to negative ions for 30 days, and the other 10 mice were used as control. The air ionizer is mounted on the inner wall of the cage. The body weight of mice was monitored daily **(A)**. Lungs of the control mice **(B)** and ion-exposed mice **(C)** were collected on day 30 and were fixed with formalin, embedded in paraffin, and stained with hematoxylin and eosin.

Six-week-old female Balb/c mice were exposed to the negative ions for 30 days. The body weight of mice was monitored daily. As shown in **Figure 7A**, the body weight of mice in the negative ion group increased gradually, and there was no significant difference in body weight compared to that of the control group (*P* > 0.05). On day 30, blood of the mice was collected for blood routine analysis. All of the blood routine indexes were within the normal range of Balb/c mice (Zou et al., 2019; Li et al., 2021), and no significant difference was found between the control group and the negative ion group (**Table S2**). The lungs of mice were fixed with formalin, embedded in paraffin, and stained with hematoxylin and eosin. The bronchial epithelial cells of the two groups of mice were closely arranged, evenly colored, and in normal shape in lung sections (**Figures 7B, C** **)**; the thickness of the alveolar wall was uniform, and no obvious thickening was observed; the morphology of the alveolar epithelial cells was normal, the size of the alveoli was uniform, and the alveoli were clean, and no obvious abnormality was observed in the tissues.

## Discussion

Controlling the source of infection, cutting off the transmission route, and protecting the susceptible population are the key measures to block the transmission of respiratory pathogens (Esike et al., 2021). In this study, the method of inactivating viral aerosol by negative ions is one of the important ways to cut off the transmission route. We chose SARS-CoV-2 and influenza A virus as test pathogens because they are important representatives of the pandemic respiratory virus of concern.

This study confirmed that air ionization is an effective means of pandemic virus inactivation. Negative ions effectively reduced the infectivity and RNA copies of fixed viruses. The disinfection efficiency was >99.8% after 1-h exposure. Moreover, negative ions could also effectively inactivate aerosolized viruses and protect susceptible animals from artificial viral aerosols. The inhalation ID_50_ of SARS-CoV-2 and influenza A virus were increased 4.44 times and 4.22 times with the use of the negative ionizer. In particular, negative ions 100% inhibited the aerosol transmission of SARS-CoV-2 and influenza A virus between infected animals and naive animals, which is closer to the authentic natural infections.

However, the inactivation mechanism against microorganisms by negative ions is still not well known. The generation of reactive radicals such as O_2_^-^ may contribute to the damage to either the protein or the nucleic acid structure of the viruses and eventual inactivation (Nishikawa and Nojima, 2003; Hagbom et al., 2015). Kim et al. (2011) used field emission scanning electron microscopy images and fluorescence microscopy images and found that electrostatic disruption of bacteria might be the dominant antibacterial effect. Digel et al. (2005) proposed that the *in situ* hydroxyl radical formation on the surface of bacteria might the leading mechanism of bacterial inactivation. Although the antimicrobial mechanism of negative ions is still controversial, these possible mechanisms all determine that the ion disinfection method has the advantage of broad spectrum. Viruses, bacteria, and fungi of any subtype, species, or variant can all be inactivated.

In addition to disinfection and purification functions, negative ions are also beneficial to human health. Lee et al. (Lee et al., 2017) found that negative ions mediated the regulation of autonomic nervous system activity and enhanced parasympathetic activity. Pino and Ragione (2013) reviewed the evidence base of negative ions in improving neuropsychological performance and treating mood disorders. Nakane et al. (2002) found that negative air ions were effective for the reduction of and the prompt recovery from stress caused by computer operation.

The negative ion disinfection method has a variety of application scenarios. In this study, we used a wearable air ionizer, which was 30–50 cm below the head when worn. It provided negative ions with the concentration of 5.61 × 10^4^~1.06 × 10^5^ ions/cm^3^ in the head area, which could not only purify air particles but also inactivate microorganisms. In addition, negative ion disinfection technology can also be made into an indoor air purifier for indoor space disinfection (Grinshpun et al., 2005) and applied for ventilation system filter disinfection (Huang et al., 2008; Hyun et al., 2017) or ventilation duct disinfection (Zhou et al., 2018; Nunayon et al., 2022) to prevent the movement of microorganisms through ventilation bulk airflows and eventual transmission.

In terms of safety, one major contentious concern about air ionizers is the production of ozone, which has strong oxidation and adversely affects the respiratory functions in humans (Castillejos et al., 1992). According to the ozone emission standard of indoor air cleaning devices issued by the California Air Resources Board (California Air Resources Board), the ozone emission concentration should not be more than 50 ppb. In this study, the air ionizer did not produce detectable levels of ozone (<1 ppb, measured by pump type ozone detector, model OZA-T30, Honri Airclean Technology Co., Ltd.), much lower than the emission standard. Moreover, BALB/c mice exposed to negative ions for 4 weeks showed no abnormalities in body weight, blood routine analysis, and lung pathology. Therefore, the effects on human of ozone emission from the air ionizer in this study are negligible.

Our study has 2 limitations. First, the inactivation mechanism against viruses by negative ions is not included in this study and needs further study. Second, the negative ionizer cannot reduce the infection rate when the susceptible animals were exposed to high viral aerosols (**Figure 5D**), which is a limitation of this strategy.

The performance of the negative ionizer compared to vaccines and wearing masks was summarized in **Table 1**. Our finding proposed that negative ions could serve as a new preventive strategy in addition to vaccines and wearing masks and make contributions in the prevention and control of respiratory infectious diseases.

**Table 1:** Performance comparison of different preventive strategies.

Preventive strategy	Performance				
	Price	Reusability	Wear comfort	Broad-spectrum antimicrobial properties	Particulate matter purification
**Negative ionizer**	Cheap	Reusable	Comfortable	High	Yes
**Vaccine**	Very expensive	Update periodically	/	Low	No
**Face mask**	Very cheap	Single use	Uncomfortable	High	Yes

## Data Availability Statement

The original contributions presented in the study are included in the article/**Supplementary Material**. Further inquiries can be directed to the corresponding authors.

## Ethics Statement

The animal study was reviewed and approved by The Animal Care and Use Committee of the Changchun Veterinary Institute.

## Author Contributions

ZG, YG, NJ, and JXL designed the project. CZ and HC performed the experiments. CMZ, ZC, XJ, JiL, ZW, and JuL analyzed the data. ZG, CZ, and HC drafted the article. NJ, JXL, and YG critically revised the article. All authors contributed to the article and approved the submitted version.

## Funding

This work was financially supported by the National Natural Science Foundation of China (82150202).

## Conflict of Interest

The authors declare that the research was conducted in the absence of any commercial or financial relationships that could be construed as a potential conflict of interest.

## Publisher’s Note

All claims expressed in this article are solely those of the authors and do not necessarily represent those of their affiliated organizations, or those of the publisher, the editors and the reviewers. Any product that may be evaluated in this article, or claim that may be made by its manufacturer, is not guaranteed or endorsed by the publisher.

## Supplementary Material

Supplementary Figure 1 Pictures of the portable and wearable negative ionizers.
