# Supplementary figures and images for: Aerosol Transmission of the Pandemic SARS-CoV-2 and Influenza A Virus Was Blocked by Negative Ions
Source: Front Cell Infect Microbiol. 2022 Apr 29;12:897416. doi: 10.3389/fcimb.2022.897416 (PMC9105223; doi:10.3389/fcimb.2022.897416)

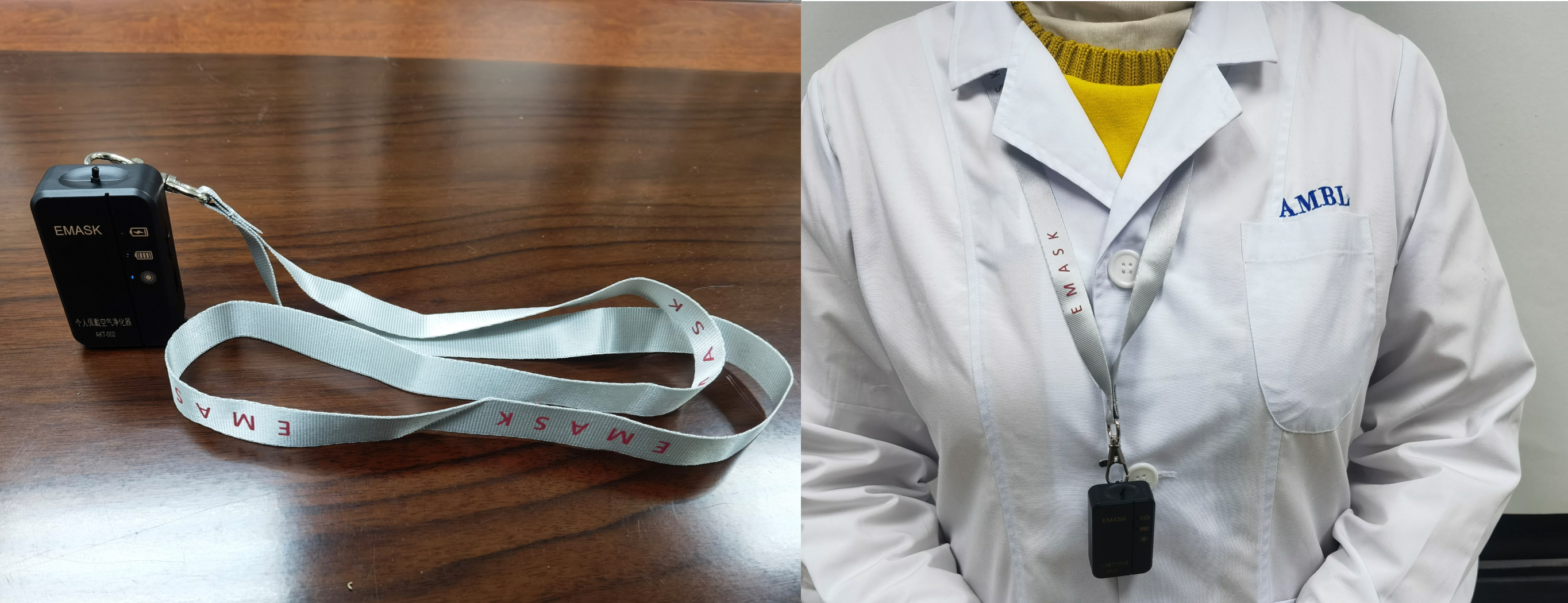

Supplement: Supplementary Figure 1 — Pictures of the portable and wearable negative ionizers. [file Image_1.tif]
