# Supplementary material for: Aerosol Transmission of the Pandemic SARS-CoV-2 and Influenza A Virus Was Blocked by Negative Ions
Source: Front Cell Infect Microbiol. 2022 Apr 29;12:897416. doi: 10.3389/fcimb.2022.897416 (PMC9105223; doi:10.3389/fcimb.2022.897416)
Supplement: Supplementary file 2 [file Table_1.docx]

**Table S1. Disinfection efficiency of negative ions on SARS-CoV-2 and influenza A virus.**

| **Virus** | **Disinfection**  **height (cm)** | **Type** | **Disinfection time (min)** | | | | |
| --- | --- | --- | --- | --- | --- | --- | --- |
|  |  |  | **5** | **10** | **20** | **40** | **60** |
| SARS-CoV-2 | 30 | Viral titer | 43.77% | 78.46% | 99.32% | 99.97% | >99.99% |
|  |  | Viral RNA copies | 30.60% | 78.87% | 98.52% | 99.97% | >99.99% |
|  | 50 | Viral titer | 0% | 43.77% | 85.32% | 99.56% | 99.98% |
|  |  | Viral RNA copies | 3.83% | 54.87% | 91.86% | 99.63% | 99.98% |
| Influenza A virus | 30 | Viral titer | 31.87% | 61.69% | 99.62% | 99.99% | >99.99% |
|  |  | Viral RNA copies | 37.66% | 59.26% | 99.72% | 99.99% | >99.99% |
|  | 50 | Viral titer | 0% | 31.87% | 94.38% | 99.90% | 99.99% |
|  |  | Viral RNA copies | 9.80% | 44.85% | 97.21% | 99.96% | >99.99% |
