# Supplementary material for: Aerosol Transmission of the Pandemic SARS-CoV-2 and Influenza A Virus Was Blocked by Negative Ions
Source: Front Cell Infect Microbiol. 2022 Apr 29;12:897416. doi: 10.3389/fcimb.2022.897416 (PMC9105223; doi:10.3389/fcimb.2022.897416)
Supplement: Supplementary file 3 [file Table_2.docx]

**Table S2.** **Blood routine results of mice exposed to negative ions for 30 days.** Ten mice were exposure to negative ions for 30 days, and the other ten mice were used as control. The air ionizer is mounted on the inner wall of the cage. Blood of the control mice and ions exposed mice were collected at day 30 for blood routine analysis

| **Item** | **Control group** | **Negative ion group** |
| --- | --- | --- |
| White blood cell (10^9^/L) | 4.8±0.3 | 4.6±0.4 |
| Lymphocyte (10^9^/L) | 3.47±0.05 | 3.52±0.14 |
| Monocyte (10^9^/L) | 0.18±0.01 | 0.18±0.02 |
| Neutrophil (10^9^/L) | 2.35±0.13 | 2.33±0.08 |
| Red Blood Cell (10^12^/L) | 9.7±0.7 | 10.1±0.5 |
| Platelet (10^9^/L) | 544±35 | 528±72 |
| Hemoglobin (g/L) | 144±18 | 147±14 |
| Hematocrit (%) | 45.07±4.32 | 44.58±2.57 |
| Mean corpuscular hemoglobin (pg) | 18.6±1.9 | 18.7±1.4 |
| Mean corpuscular Hemoglobin Concentration (g/L) | 347±5 | 349±11 |
| Platelet distributionwidth (fL) | 3.4±0.3 | 3.3±0.2 |
| Mean corpuscular volume (fL) | 45.4±0.8 | 46.5±1.8 |
| Mean platelet volume (fL) | 2.4±0.3 | 2.2±0.2 |
